# Low-dose antibiotics: current status and outlook for the future

**DOI:** 10.3389/fmicb.2014.00478

**Published:** 2014-09-10

**Authors:** Joshua D. Nosanchuk, Jun Lin, Robert P. Hunter, Rustam I. Aminov

**Affiliations:** ^1^Department of Medicine, Albert Einstein College of Medicine of Yeshiva UniversityBronx, NY, USA; ^2^Department of Microbiology and Immunology, Albert Einstein College of Medicine of Yeshiva UniversityBronx, NY, USA; ^3^Department of Animal Science, The University of TennesseeKnoxville, TN, USA; ^4^Parnell PharmaceuticalsOverland Park, KS, USA; ^5^Section for Bacteriology, Pathology, and Parasitology, National Veterinary Institute, Technical University of DenmarkFrederiksberg, Denmark

**Keywords:** antibiotics, low dose antibiotics, immunomodulatory effect, environmental impact, growth promotion, feed additives

Antimicrobial therapy is a key factor in our success against pathogens poised to ravage at risk or infected individuals. However, we are currently at a watershed point as we face a growing crisis of antibiotic resistance among diverse pathogens. One area of intense interest is the impact of the application of antibiotics for uses other than the treatment of patients and the association with such utilization with emerging drug resistance. This Research Topic “Low-dose antibiotics: current status and outlook for the future” in *Frontiers in Microbiology: Antimicrobials, Resistance, and Chemotherapy* details various aspects of the wide ranging effects of antimicrobial therapy from areas such as the regulation of host responses to modulation of bacterial virulence factors to acquisition of antibiotic resistance genes.

A remarkable and often overlooked fundamental of antibiotics is that they have biological activities beyond microbial killing. The host modulatory aspects of macrolides, tetracyclines, and beta-lactams are reviewed by Aminov (2013a) underscoring how, for example, macrolides such as azithromycin are routinely used for immunomodulation in patients with chronic pulmonary disease rather than for an antimicrobial effect. Azithromycin is also used as a tool by Imperi et al. to detail how non-conventional thinking about regulating virulence factors or modifying host inflammatory cascades are useful to combating major pathogens such as *Pseudomonas aeruginosa* (Imperi et al., 2014). Along this line, Morita and colleagues carefully detail the pleotropic responses of *P. aeruginosa* to sub-therapeutic levels of several antibacterials and propose avenues to pursue to combat this pathogen, such as developing efflux pump inhibitors (Morita et al., 2014). In their article, Charlebois et al. show *Clostridium perfringens* biofilm can be regulated by certain antibiotics at low concentrations (Charlebois et al., 2014). For example, low dose bacitracin significantly enhances biofilm formation whereas low dose penicillin reduces biofilm. This work underscores how there are untoward effects that are not predictable when antimicrobials are administered at low concentrations. Providing a view on specific host effector pathways with antimicrobials, Mihu et al. detail how antifungal medications effectively stimulate host responses via engagement with toll-like receptors (Mihu et al., 2014). In light of the expanding difficulties with drug resistance and a lack of therapeutics to combat them, Clark presents a cogent call for pursing Ca^2+^ modulating strategies where by host Ca^2+^ homeostasis is modulated to block pathogens from effectively utilizing this essential element (Clark, 2013).

An important focus in this Research Topic is the use of antibiotics as growth enhancers in animals. Sorensen and colleagues provide key insights into the effects of scientific evidence on the policy decisions on the use of low-dose antimicrobials in livestock for growth promotion and disease prevention particularly delineating how data have led to the European Union's ban of low-dose antimicrobials whereas their use in the United States of America remains in flux (Sorensen et al., 2014). The bottom line is that there is an urgent need to develop policy based on well derived data, with this data being easily and widely available to independent parties. The articles by Cheng et al. (2014), Chattopadhyay (2014), and Hao et al. (2014) all further underscore critically important facets of the continued utilization of antibiotics in animal husbandry. Looft and colleagues detail their research on how the use of the in-feed antibiotic carbadox cases dramatic short- and long-term effects on the composition of porcine gut microbiota (Looft et al., 2014). Diarra and Malouin specifically describe the impact of antibiotics in Canadian poultry production and describe the use of alternatives, such as bioactive molecules from cranberries, that should not drive antibiotic resistance (Diarra and Malouin, 2014). Similarly, Rendondo et al. provide thoughtful insights into the use of tannins in lieu of antibiotics for improving health in poultry (Redondo et al., 2014). Lin details that the effective of antibiotics as growth promoters is linked to decreased activities of bile salt hydrolase, which thus makes targeting this enzyme directly a promising method for removing antibiotics for use as growth enhancers (Lin, 2014).

You and Silbergeld critically discuss the effects of antimicrobials as drivers of resistome expansion (You and Silbergeld, 2014), a major secondary effect due to environmental pollution. The effects of antibiotics permeating our environment are highlighted by Conro and colleagues who present their findings that the presence of antibiotics in aquatic environments can induce co-aggregation of bacterial species as an effective mechanism to combat the effects of the antimicrobials (Corno et al., 2014), which can lead to extensive resistance through the transfer of resistance genes among these aggregated bacteria. It is a small leap for these microbes to then impact humans and other organisms. Aminov provides the example of the rampant use of tetracyclines for non-medical purposes as driving the penetration of *tet*(X) into pathogenic microbial communities (Aminov, 2013b). Chowdhury and colleagues eloquently discuss the import of surveillance strategies for critically elucidating the emergence of drug resistant pathogens in the context of low-dose antibiotic use in animal husbandry (Roy Chowdhury et al., 2014).

In summary, the articles within this Research Topic serve as a “call to arms” for scientists, policy makers and the public to be increasingly vigilant about the use of antimicrobials, particularly in low-dose or where they can become widespread in the environment, in order to maintain our capacity to effectively care for individuals with infectious diseases. The articles also provide new concepts for approaches for the development of antimicrobials as well as for novel growth enhancers for the use in animal husbandry.

## Conflict of interest statement

The authors declare that the research was conducted in the absence of any commercial or financial relationships that could be construed as a potential conflict of interest.
